# Global educational initiative for managing major depressive disorders in primary care: the MDD minds project

**DOI:** 10.3389/fmed.2025.1679133

**Published:** 2025-11-27

**Authors:** Christopher Dowrick, Mary Ales, Abdullah AlKhathami, Dewanto Andoko, Adekunle Joseph Ariba, Ryuki Kassai, Cindy Lam, Christos Lionis, Joy Mugambi, Sankha Randenikumara, Shelly Rodrigues, André Tavares, Ana Bertha Perez Villalva

**Affiliations:** 1 Department of Mental Health and Primary Care, University of Liverpool, Liverpool, United Kingdom; 2 Mosaica Solutions LLC, Madison, WI/Kansas City, MO, United States; 3 Postgraduate Medical Education, Dammam, Saudi Arabia; 4 Faculty of Medicine, Pelita Harapan University, Tangerang, Indonesia; 5 Department of Family Medicine, Olabisi Onabanjo University Teaching Hospital, Sagamu, Nigeria; 6 Department of Community and Family Medicine, Fukushima Medical University, Fukushima, Japan; 7 Department of Family Medicine and Primary Care, School of Clinical Medicine, The University of Hong Kong, Hong Kong, Hong Kong SAR, China; 8 Laboratory of Health and Society, School of Medicine, University of Crete, Crete, Greece; 9 Department of Psychology, University of Limassol, Limassol, Cyprus; 10 Kenya Association of Family Physicians, Nairobi, Kenya; 11 College of General Practitioners of Sri Lanka, Colombo, Sri Lanka; 12 Faculty of Medicine, University of Porto, Porto, Portugal; 13 Institute of Social Security and Services for State Workers (ISSSTE), Family Medicine Clinic, Mexico City, Mexico

**Keywords:** depression, primary care, family doctors, online training, Train-the-Trainer, screening

## Abstract

Depressive disorders are common and disabling, and a substantial treatment gap exists particularly in low-resource settings. Family doctors are well-placed to bridge this gap but often lack the confidence, knowledge and skill to do so. The World Organization of Family Doctors (WONCA) and its collaborators designed the MDD Minds project to improve care for major depressive disorders delivered by family doctors in Africa/Middle East (Kenya, Nigeria, and Saudi Arabia), Asia (Indonesia, Japan, and Sri Lanka) and Latin America (Brazil, Mexico, and Peru). The MDD Minds organizational structure included a steering committee, a design and measurement team and a group of master faculty composed of primary mental health care experts from participating countries. Project delivery was in three phases: MDD Minds 101, an online programme with seven modules; Train-the-Trainer, a group-based approach combining online and real-time elements and leading to delivery of in-country educational activities; and Performance- in-Practice, focused on enhanced depression screening for patients with diabetes. Recruitment was conducted by WONCA in collaboration with national primary care organizations. Project delivery began in October 2023 and was completed in December 2024. Enrollment and completion rates were tracked: 2,892 family doctors enrolled in MDD Minds 101; 581 (20%) completed the course. In Train-the-Trainer 210 scholars enrolled; 126 (60%) completed the program, delivering 143 educational sessions to 1,697 other healthcare professionals in their respective regions. Nine primary care teams in Brazil, Kenya, Japan and Nigeria participated in the Performance-in-Practice program; among 1,592 diabetes patients screened, 26.6% were diagnosed with depression and promptly managed. Strategic alignment between partnering organizations, in combination with world-wide expertise in mental health, ensured effective participation. Language and cultural adaptation were important to serving the diverse range of learners, as was ease of access to online platforms and enhanced use of social media. Our online course completion rate exceeded our expectations. The MDD Minds project demonstrates WONCA’s ability to deliver high-quality educational programming at scale, as well as the benefits of localized frameworks that support family doctors, primary care teams, and patients in improving the care of those with major depressive disorder. It offers a replicable and sustainable approach to enhancing mental healthcare in diverse primary care settings.

## Introduction

Mental health is a fundamental pillar of global health, yet depressive disorders remain prevalent and disabling conditions. In 2019, 275 million cases of major depressive disorder were diagnosed across the globe, an increase of over 100 million from 1990. The highest rate of increase is in low-resource settings (1), where the burden of illness is greatest for women (2). In 2021 depressive disorders accounted for 56 million years lost to disability, 36.5% more than in 2010 (3).

The consequent decrease in productivity takes a financial toll, with the global cost estimated at $1 trillion annually. In high-income countries, about half of people suffering from depression are not diagnosed or treated appropriately, and the percentage increases to between 80% and 90% in low-income countries (4).

Most patients with mental health problems report first to their family doctors. The integration of mental health into primary care, especially into family medicine, is recognized as a priority by international agencies (5, 6). Optimal mental health outcomes are achieved through integration of mental health services into the general framework of existing primary health care (7). This strategy has the potential to reduce stigma, protect patients’ rights, improve social integration, reduce chronicity, improve human resource capacity for mental health and ultimately, access to effective mental health care (8).

The need to train family doctors to acquire current evidence-based knowledge, skills and practices of mental health care at the primary care level is urgent. This training supports family doctors so they can deliver non-stigmatizing mental care and help reduce the treatment gap. Clear evidence and practical guidance are available to empower family doctors with the skills necessary to diagnose and treat major depression (9).

It is imperative to translate this robust evidence base into practice through context- sensitive interventions. When primary care providers such as family doctors are enabled through training, they effectively deliver mental health care, including application of appropriate psychotherapy (10, 11).

Delivery of effective care for patients with major depressive disorders (MDD) also requires systems empowerment for family doctors. Family doctors not only need to develop their own knowledge and skills in the diagnosis and management of major depression, they also need to be empowered to work as part of a team with local communities and families, and to advocate for practice transformation and integrated care (12).

Interaction and discussions are necessary to improve communication skills related to mental health issues. The constraints imposed by the COVID-19 pandemic and the needs of a wide geographical spread of participants have led to an increased emphasis on online learning. When these include a combination of live sessions with faculty, online presentations and webinars, with access to reading materials and videos, they are valuable formats for successful educational delivery (13, 14).

Despite numerous local training initiatives, no previous training has globally addressed MDD training across diverse cultural contexts using a standardized yet adaptable curriculum at this scale.

In this paper we describe the context for the MDD Minds project, explain its content and structure, provide an overview of results, and consider the implications for best practice for international educational interventions in primary care mental health.

Specific objectives of the project were to increase family doctor competence to:

- Diagnose MDD in context of cultural variations in presentation;
- Educate patients in health promotion and lifestyle change;
- Employ shared decision-making;
- Select appropriate pharmacologic and non-pharmacologic therapy, including group interventions;
- Manage co-morbidities of MDD, including cardio-vascular disease and diabetes;
- Develop team approaches, including family and community support;
- Communicate with specialists in making referrals;
- Follow-up with patients to reevaluate care; and
- Train other primary care practitioners in diagnosis and management of MDD.

## Context

**FIGURE 1:**
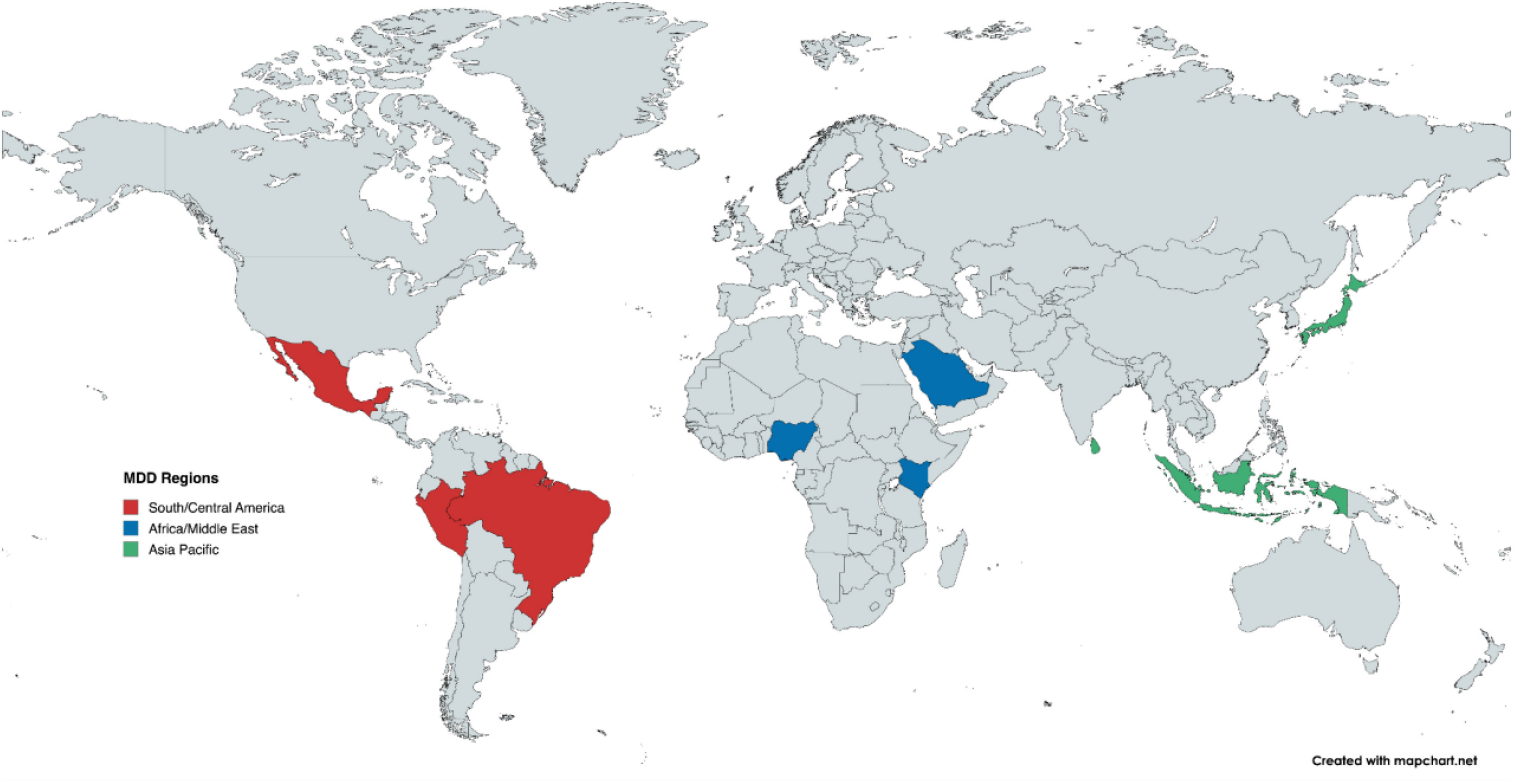
Map of participating countries, by region.

The WONCA MDD Minds project was delivered in nine countries across Africa/Middle East, Asia- Pacific, and Latin America: Brazil, Indonesia, Japan, Kenya Nigeria, Mexico, Peru, Saudi Arabia, and Sri Lanka (see Figure 1). These countries were selected for their geographic diversity, varied economies, practice settings, and their leadership in family medicine.

In *Africa*, despite the high prevalence of mental disorders the mental health gap remains enormous, up to 85% in some countries (15). Across the continent, typically less than 1% of the health budget is spent on mental health (16), and most of this limited resource is expended on tertiary mental health institutions whose services are inaccessible to the majority of patients with mental health problems. The health systems in Africa are still burdened with large volumes of communicable diseases including Malaria, HIV/AIDS and the COVID-19 pandemic, and these compete better than mental health problems for the meager resource available for health care. Importantly, the stigma associated with mental conditions is extremely high and constitutes a major impediment to seeking care.

In the *Asia-Pacific* region, a needs assessment survey of more than 300 WONCA members demonstrated a high need for education related to mental health issues. Interviews with family doctors in that study concluded that improving communication skills was a priority: communicating with patients requires specific skills to set expectations, explain disease processes and recommend therapy. Additionally, a need for skills in communicating with specialists to gain trust and respect from the specialists was expressed by many family doctors (17). During 2018 and 2019, WONCA delivered three train-the-trainer programs for family doctors in the Asia-Pacific Region in the assessment and management of depression and anxiety in primary care. This led to a training cascade with primary care educational events hosted in participating doctors’ home countries, including China, Japan (14), Nepal (18), and Vietnam.

In *Latin America*, despite the existence of specialized and specific training in family and community medicine, many curricula do not include training on communication skills and psychosocial interventions. In Brazil only 22% of patients with depression received treatment, usually pharmacological interventions (19, 20), and low levels of competence were identified during training with the WHO mhGAP Intervention Guide (21). During 2022 Mosaica Solutions, with WONCA and in-country champions, undertook an extensive needs assessment on issues related to mental health care provided by family doctors. The assessment, completed in Brazil, Chile, Colombia, Mexico and Peru provided clinical and gap data to support training for family and primary care physicians, as well as an educational construct to support learning.

## Key project elements

Major depressive disorders Minds is a three-phase educational project for family doctors in the diagnosis and treatment of major depressive disorders (MDD) in primary healthcare settings. The first phase is a set of online modules - MDD Minds 101 - followed by a Train-the-Trainer phase and a Performance in Practice component.

Members of the WONCA Working Party for Mental Health undertook preparatory work during 2020 and 2021 in setting the parameters for the MDD Minds program including three expert panel meetings for Asia, Latin America and Africa/Middle East. Project delivery began in October 2023 and was completed in December 2024.

### Project content and structure

Guided by senior members of the WONCA Working Party for Mental Health, the MDD Minds curriculum was developed during 2023 by the Master Faculty of mental health experts from 12 countries across Latin America, Europe, Africa, the Middle East, and Asia. The curriculum was tailored to the specific needs and cultural contexts of each region and country, enhancing both relevance and applicability.

*MDD Minds 101* is a series of seven fully accessible online modules covering the following topics:

1. Introduction and the burden of depression
2. Starting treatment
3. Non-drug interventions
4. Pharmacotherapy
5. Managing co-morbidities
6. Follow-up and referral
7. Self-care and wellness

The modules were designed to provide learners with clinical information and practice tools to enhance their care of patients with MDD (22). The case-based content was developed by Mosaica Solutions building on curriculum developed in the Asia-Pacific Train-the-Trainer project (referenced above), with review by WONCA experts and oversight by the Steering Committee. The modules were presented in video format, each averaging 15–20 min, with resources for further learning provided to augment the sessions. Post-session reflection questions were asked as learners moved through the modules and each module included a quick review of the previous session. The modules were presented in English. Japanese, Portuguese, and Spanish versions were subsequently developed with the assistance of local faculty. Those completing the MDD Minds 101 curriculum received a bonus module that included resources from the World Health Organization (WHO). Expected participation for MDD Minds 101 was 750 family doctors (250 from each region).

Family doctors from the nine participating countries were recruited via email, web notices, social media (including WhatsApp) and organizational invitations. The following national WONCA membership organizations assisted with the recruitment process:

- Brazil: Sociedade Brasileira de Medicina de Família e Comunidade (SBMFC)
- Indonesia: Indonesian Association of Family Physicians
- Japan: Japan Primary Care Association
- Kenya: Kenya Association of Family Physicians
- Mexico: Federación Mexicana de Especialistas y Residentes en Medicina Familiar
- Nigeria: Society of Family Physicians of Nigeria
- Peru: Sociedad Peruana de Medicina Familiar y Comunitaria
- Saudi Arabia: Saudi Society of Family and Community Medicine
- Sri Lanka: College of General Practitioners of Sri Lanka

The World Organization of Family Doctors and the Master faculty remained in contact with learners throughout the engagement to support completion and next steps. Enrollees in in the 101 program were recruited through their WONCA Member country organizations. Once enrolled, learners were encouraged to complete the 101 programme via email reminders from WONCA and master faculty members. Participants in the Train the Trainer program were recruited from MDD Minds 101 completers as well as through personal recommendations from faculty and physician leaders.

The Train the Trainer and Performance in Practice faculty were provided with curriculum resources and encouraged to adapt these tools based on local practice norms and country localization therefore we did not require fidelity to the provided curriculum.

Scholars who completed MDD Minds 101 were eligible to enter the *Train-the-Trainer* (TtT) component of the program. The TtT course, led by local Master Faculty, combined online content with real-time sessions with other scholars.

The course reinforced the content areas from MDD Minds 101, adding application in practice and skill-building. There were additional modules on motivational interviewing, communication skills and presentation training. The TtT course used Trello (a visual project management and collaborative tool), Zoom and WhatsApp for engagement. Master Faculty members were encouraged to add and translate content as needed. For example, the Saudi group enhanced patient interviews through the implementation of the AlKhathami Five-Step approach (23). Scholars participated in four sessions with Master Faculty and their cohort colleagues. These included one “Scholars” Choice’ session designed to meet expressed localized needs. Each scholar was required to deliver at least one local training session to disseminate knowledge. Expected participation for the TtT component was 150 family doctors (50 per region).

For the *Performance-in-Practice* (PiP) component Master Faculty aimed to recruit two practices from one country in each of the three regions. The focus was on implementing a systematic approach for depression screening amongst adult patients with diabetes, a long-term disease commonly diagnosed across primary care internationally and associated with increased risk for depression (24). Practices were invited to evaluate the patient’s record for a previous diagnosis of depression; and then implement a screening process aligned with the practice needs (25, 26).

The PiP project included a two-cycle improvement process with baseline measurement and final process cycles. Coaching by the Master Faculty and education on elements of performance and quality improvement (QI) were provided through four virtual calls scheduled to align with the data collection and reflection process. Learners were encouraged to interact using the Trello platform that housed the native language videos, resources and localized content including practice data. The 12-week project was designed into four phases. The practices completed “swim lane” analysis and Plan-Do- Study-Act planning prior to beginning their improvement cycles (27). Practices received a participation stipend to support the time commitment to the QI project. Both TtT and PiP were completed in December 2024.

**FIGURE 2:**
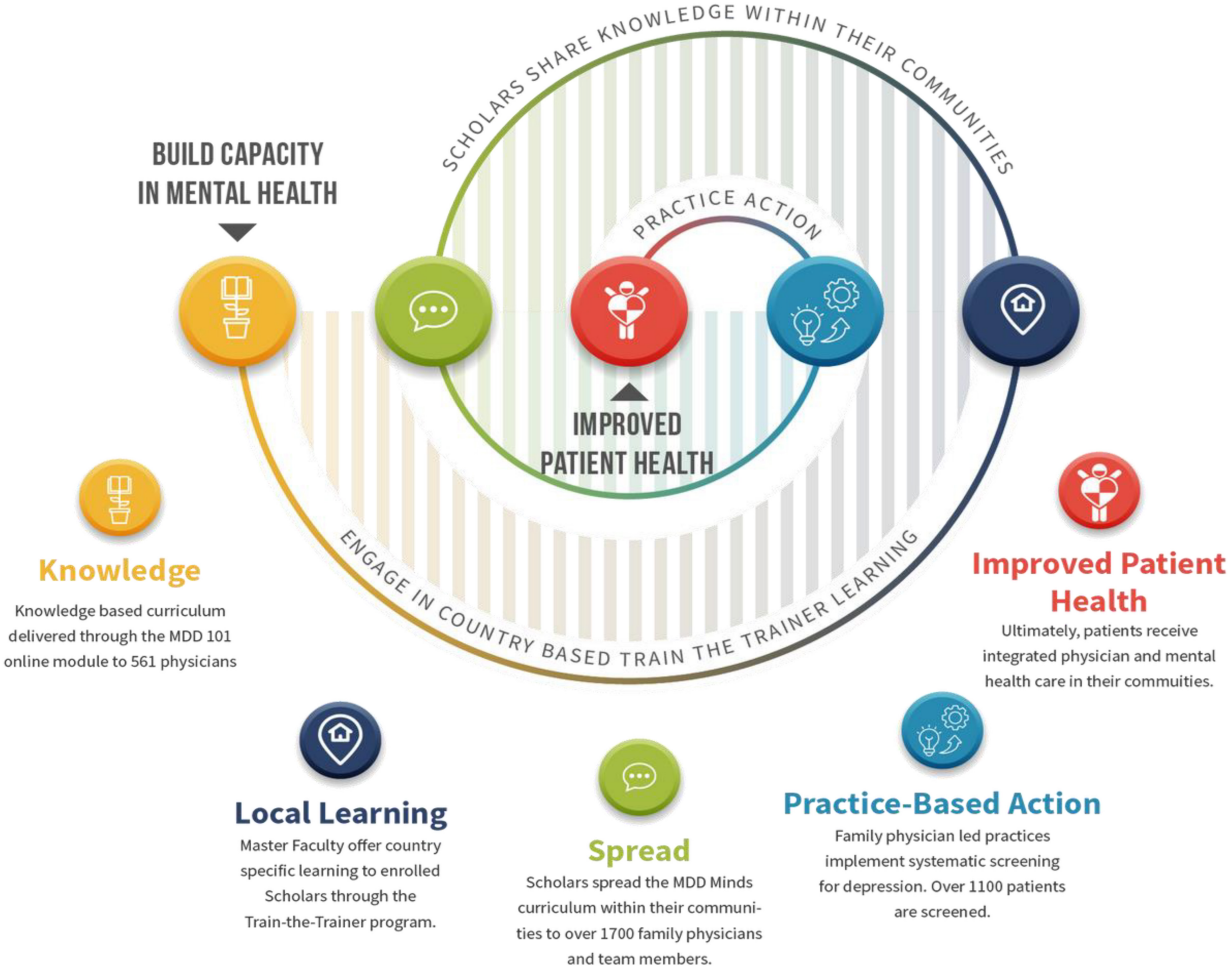
Structure of MDD minds projecture 3.

The overall structure of the MDD Minds project is summarized in Figure 2:

Each phase of the project included a comprehensive *evaluation* component. The Design and Measurement group used a variety of evaluation tools, including pre-assessment questionnaires, pre-post testing, and a series of surveys to measure the results and outcomes for the individual phases, as well as the overall project. Evaluation of the TtT phase included each scholar’s overall experience, community meeting feedback and the content and experiences of the community learners. Additionally, the Master Faculty completed an evaluation of their involvement in the overall project.


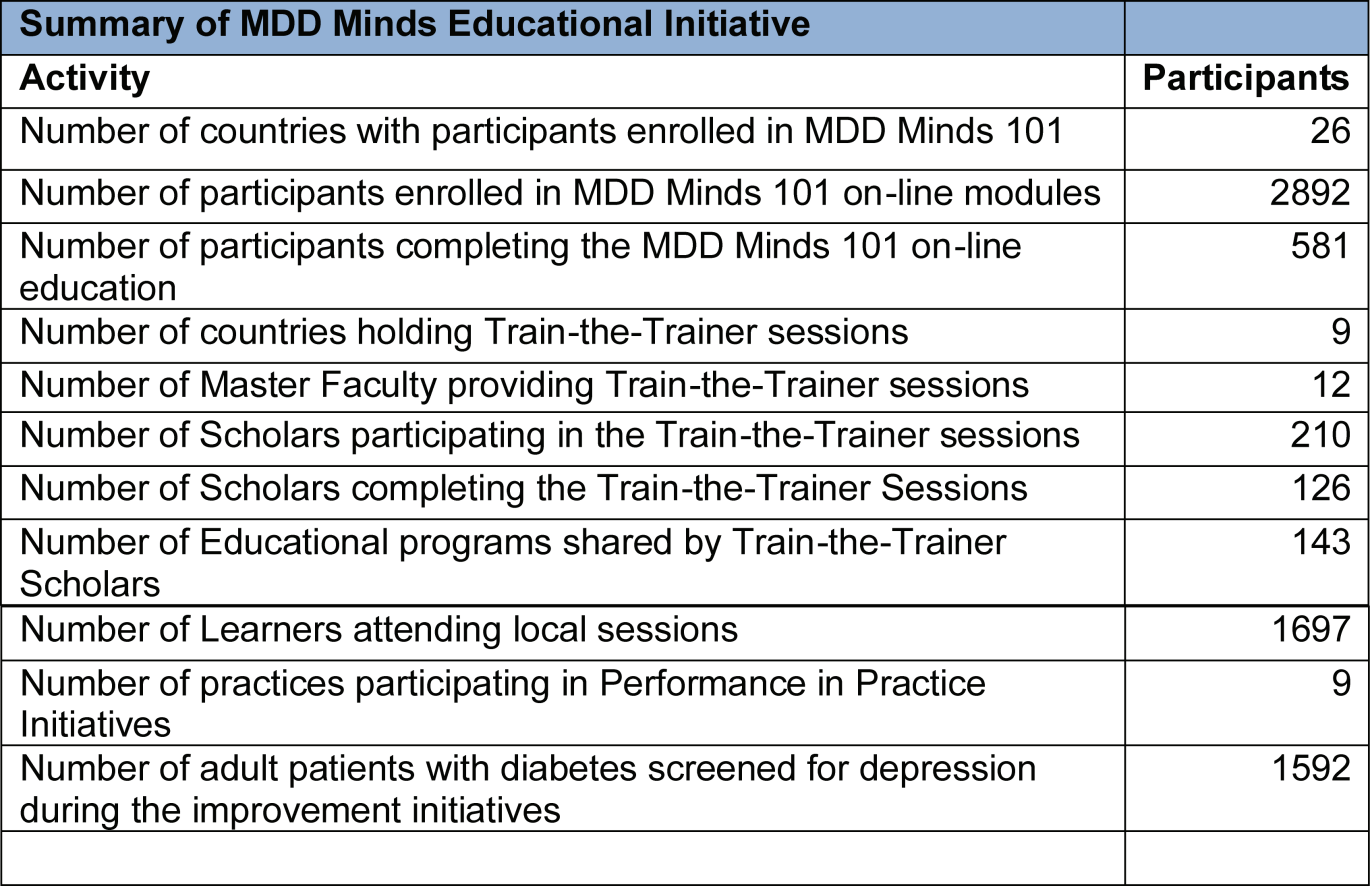


### Project management

The project benefited from a multi-level management structure, with implementation led by three working teams in addition to the internal project management coordinated by WONCA staff.

- The *Steering Committee*, which met monthly throughout the life of the project, was responsible for project oversight, support of the WONCA staff, input on the curriculum and content, and communications with the Master Faculty and participants.
- The *Design and Measurement* group, led by Mosaica Solutions, was responsible for the overall content design, development and outcomes strategies for the project, including guiding the phasing, clinical modifications, recruitment and communications; designing the dissemination plan; and analyzing the outcomes data.
- The *Master Faculty*, including representatives of each of the nine participating countries, played an integral role in the MDD Minds project. They served as content developers, translators/interpreters, teachers, coaches and advocates. Their engagement was crucial to the project’s success. Through the evaluation process they shared their successes and challenges as well as the impact this project had on their own practices. Four Master Faculty members (from Brazil, Japan, Kenya and Nigeria) both facilitated the TtT program and served as coaches for PiP.

Prior to the launch of each phase of the MDD Minds project a series of training sessions for the Master Faculty was held, ensuring they had a full understanding of the materials and implementation plans, and the opportunity to engage in conversations with each other. Master Faculty members were encouraged to localize content to meet the needs of the family doctors in their region. Support for the Master Faculty was also provided by Mosaica Solutions for the TtT and PiP components, with participation in the zoom sessions and one-on-one guidance as needed.

## Discussion

### Summary of results

We begin this section with a brief summary of the results of the MDD Minds project. These will be described and discussed in greater detail in subsequent publications.

MDD Minds 101*:* A total of 2,892 learners enrolled, with 581 participants (20% completion rate). The curriculum significantly increased participant confidence in managing depression. A total of 566 participants completed both pre- and post-training assessments. Confidence in managing depression increased from 2.97 to 4.11 on a five-point Likert scale. This improvement was statistically significant (*p* < 0.001), with a large effect size (Cohen’ *d* = 1.17), indicating a substantial gain in participant confidence following the training.”

Train-the-Trainer*:* A total of 226 scholars in nine countries enrolled in our advanced program with at least 126 (56%) completing the program. These scholars demonstrated remarkable commitment by delivering 142 educational sessions to 1,697 additional healthcare professionals in their communities.

Performance-in-Practice: Nine practice teams across Brazil, Japan, Kenya, and Nigeria implemented systematic depression screening for patients with diabetes in their practices. Through this quality improvement initiative 1,592 diabetic patients were screened with 423 (26.6%) identified as having depression.

The MDD Minds Project met all the objectives set out in our proposal, addressing critical gaps in depression care. Participating clinicians reported substantial improvements in their ability to:

- Diagnose MDD in culturally diverse contexts
- Educate patients on health promotion and lifestyle changes
- Apply shared decision-making principles
- Select appropriate pharmacological and non-pharmacological treatments
- Manage co-morbidities
- Develop team approaches to care
- Communicate effectively with specialists
- Implement activities to drive practice change, and
- Follow up with patients to review healthcare needs.

Importantly, the project also highlighted the need for healthcare provider *self- care.* This valuable outcome has strengthened resilience among participating clinicians, enabling them to better serve their patients while maintaining their own wellbeing.

### Recommendations for best practice

Best practices were identified by reviewing evaluations from the master faculty and subsequent review by the MDD Minds Steering Committee.

*Language and cultural adaptation* are necessary to serve the needs of a wide range of learners. Translating the educational material from English into Portuguese, Spanish, and Japanese was fundamental, while having further languages available may have increased engagement and completion. Cultural personalization is critical since mental health problems are addressed and managed differently across the globe: what is managed by family doctors in one country may be the purview of only psychiatrists in another country. Understanding these cultural and structural nuances is key to implementing a curriculum that improves care and patient health outcomes.

The support and engagement of *in-country organizations* was key to the recruitment process, providing another voice and invitation to local family doctors. In addition, we found the use of *social media* including WhatsApp to be both practical and cost-efficient in facilitating effective communication among the participants and Master Faculty.

Adequate time should be allowed for the development of *educational content* and material especially when local language adaptations are involved. Time should also be factored in for field testing to ensure accuracy and easy navigation of the chosen online platform.

Our *completion rate* of 20% for the MDD Minds 101 online module was higher than anticipated. The percentage rate for completion of online courses is usually between 5% and 15%, while for Massive Open Online Courses (MOOCs) it is only 5%–8% (28). The completion rate for the train-the-trainer program was in line with similar activities in primary care in Latin America (29). We recommend our practice of designing brief (15–20 min) online modules, to encourage learners to stay engaged. It is also important to ensure minimal delay between registration and course participation, as we have received feedback that technical delays in enrollment confirmation can serve as barriers to completion rates.

*Tracking data* on responses and results for each phase and from all participants is most effective when data collection methods are confirmed before the start of the initiative. Prior agreement on the use of a single data- collection platform is also recommended.

Flexibility is needed when selecting the most appropriate *in-country leader*. Physicians are busy, schedules change, life happens; it may be necessary to identify additional in- country experts to take leadership roles after the project begins. This reflects a common reality in the field. However, it is important to balance flexibility and task-sharing with continued efforts to center and empower primary care physicians in leadership roles.

## Conclusion

The MDD Minds project demonstrates the capacity of WONCA and its partners to deliver high-quality educational programming at scale, work in partnership with other international organizations, while meeting defined objectives and producing meaningful outcomes. It also highlights the benefits of localized frameworks that support family doctors and primary care teams in improving the care of patients with major depressive disorders.

The project has equipped family doctors with essential knowledge and skills to address depression, a leading cause of global disability. Most importantly, their patients have benefited from improved recognition, understanding, and treatment of their condition, with positive impacts on their mental health and wellbeing.

The potential to package MDD Minds and spread its usage is significant. The curriculum, tools and resources can be made available to other countries, with a framework for recruitment, master faculty engagement, content translation (as needed), technology recommendations, and communications provided to champions in selected locations or organizations. Considering the multimorbidity of depression with other non- communicable diseases, the MDD Minds framework may also serve as a useful template to design projects targeting other important clinical and public health topics.

As depression continues to affect hundreds of millions globally, the WONCA MDD Minds project provides a scalable and sustainable model which is adaptable to address other mental health issues or other public health priorities.

## Data Availability

The raw data supporting the conclusions of this article will be made available by the authors, without undue reservation.
